# Alcoholic foamy degeneration: an unusual presentation of the alcoholic liver disease diagnosed on autopsy

**DOI:** 10.4322/acr.2023.446

**Published:** 2023-10-11

**Authors:** Rashmi Joshi, Mayur Parkhi, Anjali Gupta, Terence Susngi, Ashwani Kumar, Deba Prasad Dhibar, Suvradeep Mitra

**Affiliations:** 1 Post Graduate Institute of Medical Education and Research, Department of Histopathology, Chandigarh, India; 2 Post Graduate Institute of Medical Education and Research, Department of Internal Medicine, Chandigarh, India

**Keywords:** Liver Diseases, Alcoholic, Pancreatitis, Acute Necrotizing, Autopsy

## Abstract

Alcoholic foamy degeneration (AFD) is an uncommon presentation of alcoholic liver disease (ALD) with characteristic histologic findings of foamy-looking hepatocytes due to the presence of abundant microvesicles of fat within the cytoplasm predominantly in perivenular and midzonal regions without inflammation and fibrosis. It is underdiagnosed as the patients quickly recover after alcoholic abstinence and are rarely caught on biopsies. AFD has better prognosis than alcoholic hepatitis, and the injury mechanism is different, warranting a different diagnosis. We present an uncommon case of AFD incidentally diagnosed during autopsy in a chronic alcoholic and diabetic man.

## INTRODUCTION

Alcoholic liver disease (ALD) evolves from steatosis to alcoholic hepatitis and cirrhosis. Usually, patients with steatosis are asymptomatic. Notwithstanding, hepatic insufficiency ensues in a later stage of the hepatic injury. However, patients with only steatosis rarely present with liver insufficiency features. This encompasses two uncommon entities, including alcoholic foamy degeneration (AFD) and alcoholic fatty liver with jaundice (AFLJ). Uncommonly, AFD presents incidentally or with nonspecific features. Acute pancreatitis is a known complication of chronic alcoholism. We report a case of a chronic-alcoholic man who succumbed to acute pancreatitis and was incidentally diagnosed with AFD on autopsy.

## CASE REPORT

A 46-year-old non-smoker with chronic alcohol use disorder and diabetic man presented to emergency with complaints of acute-onset, epigastric dull aching abdominal pain and multiple episodes of non-bilious vomiting for 1 day accompanied by decreased urine output and constipation. There was no history of hematemesis, melena, hematochezia, jaundice, or altered mental status. He had been drinking alcohol for 15 years and had a binge alcoholic consumption one day prior to the symptoms’ onset. On examination, there was epigastric tenderness without visceromegaly or icterus. There were no stigmata of chronic liver disease like clubbing, gynecomastia, collateral circulation, ascites, sarcopenia, testicular atrophy, or skin bruise. Limited investigations before his demise showed a total leukocyte count of 23,700/mL, (reference range [RR]; 4000-11000/mL), random blood sugar 303 mg/dL (RR; less than 200 mg/dL), serum amylase 215 U/L (RR; 30-110 U/L), and severe metabolic acidosis with pH 7.21, bicarbonate 8.2 mmol/L, and lactate 11.6 mmol/L. Liver function tests and other biochemical and serological tests were not performed as the patient was referred to our institute in shock and had only 9 hours stay at our institute before his demise. Electrocardiography showed sinus tachycardia. Ultrasonography highlighted a bulky, edematous, and obscured pancreas with a grade 1 fatty liver. No focal lesion or intrahepatic biliary radical dilatation was noted. The liver span was 15.6cm. The patient did not respond to the fluid, inotrope, and antibiotic therapy and succumbed to the illness only 9 hours after the admission. A complete autopsy (thoracoabdominal with brain) was performed with the clinical diagnosis of acute pancreatitis and refractory shock.

## AUTOPSY FINDINGS

**Figure 1:**
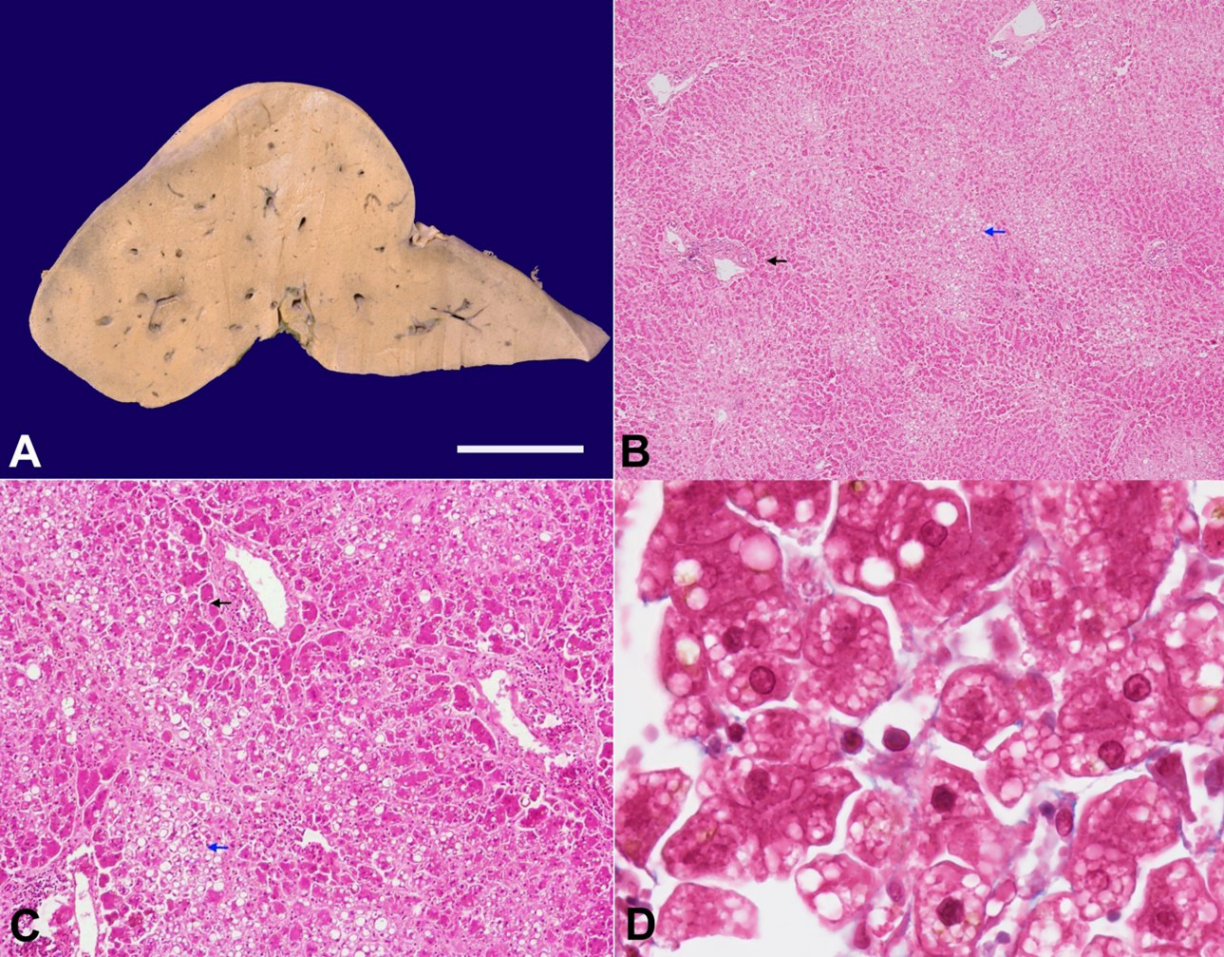
**A -** gross photograph of the liver showing a homogeneous yellow cut surface; **B** and **C -** the microphotograph showing paler zone 2 and zone 3 (blue arrow) and more eosinophilic zone 1 (black arrow) (B - H&E, x40; C - H&E, x200); **D -** the centrizonal hepatocytes are studded with microvesicles of fat, giving them a foamy appearance (Masson Trichrome, x1000).

The pancreas showed near-total hemorrhagic necrosis with relative preservation of the pancreatic head and saponification of the peripancreatic fat. There was no fibrosis in the relatively-preserved pancreatic parenchyma to suggest any element of background chronic pancreatitis. The liver was enlarged and weighed 1720gm (RR; 1200-1500gm). The hepatic capsular surface was unremarkable, and the cut surface was homogeneous yellow in color, smooth, and greasy to feel. There was no nodularity, capsular wrinkling, or space-occupying lesion (Figure 1A). On microscopic evaluation, the lobular architecture was maintained. The centrizonal (both zone 3 and zone 2) hepatocytes appeared paler as compared to the periportal (zone 1) due to the presence of a foamy appearance in the former (Figure 1B).

**Figure 2:**
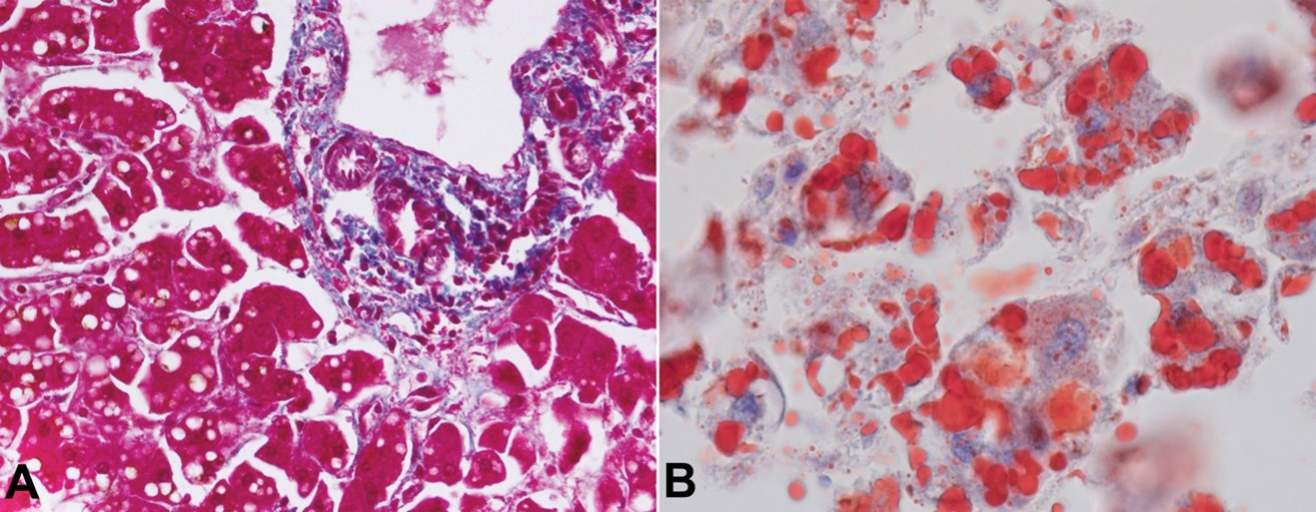
**A -** the periportal hepatocytes had eosinophilic cytoplasm with scattered macrovesicular steatosis (Masson Trichrome, x400); **B -** the presence of fat within the microvesicles is highlighted by the Oil-red-O stain (x400).

This foamy appearance in the centrizonal hepatocytes was attributed to multiple small vacuoles of fat (microvesicular steatosis) admixed with macrovesicular steatosis (Figure 1C and 1D). In contrast, the periportal hepatocytes had eosinophilic cytoplasm with scattered macrovesicular steatosis (Figure 2A). The intracytoplasmic fat was highlighted by an Oil Red O stain (PAS negative) (Figure 2B).

**Figure 3:**
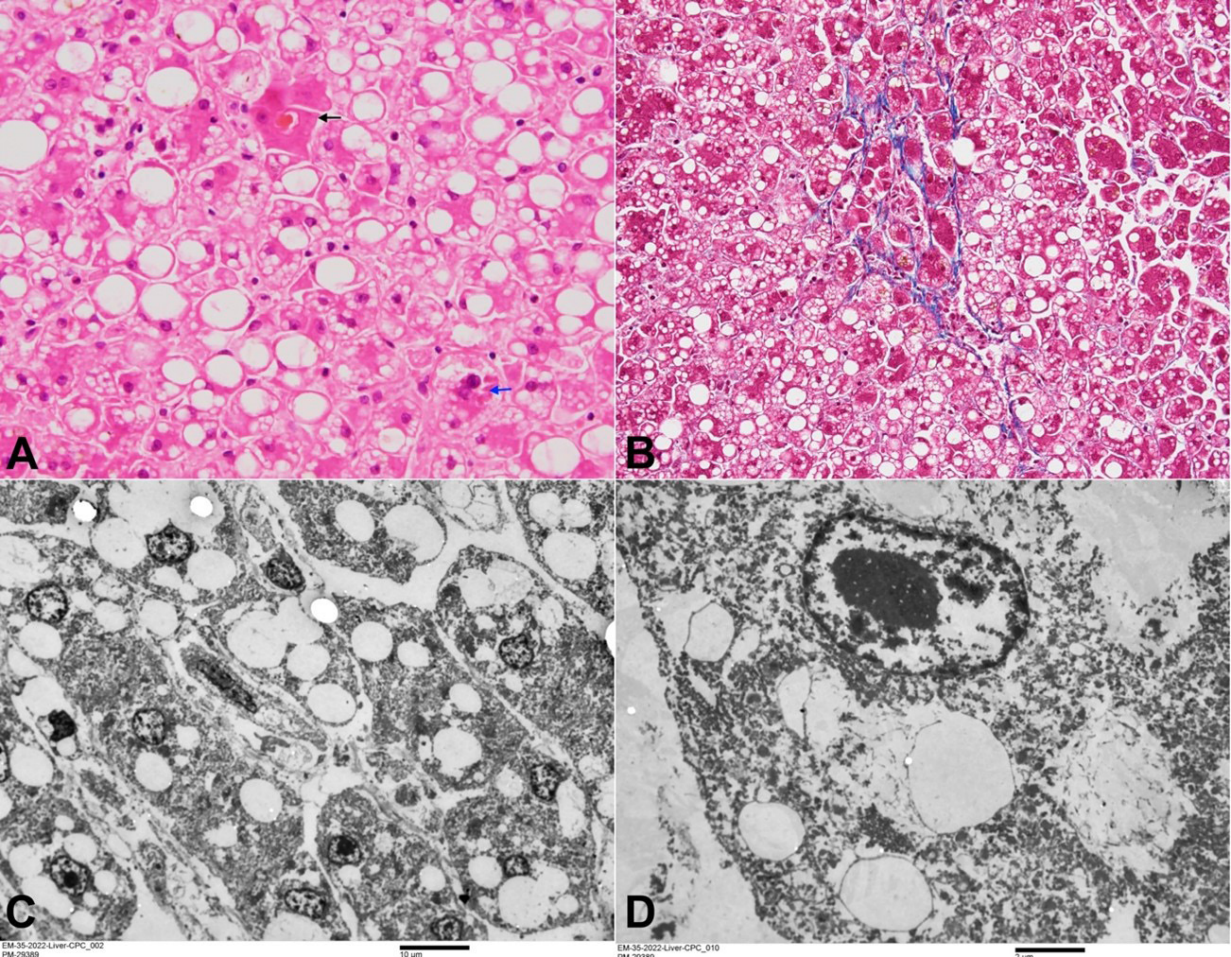
**A -** microphotograph showing occasional intracanalicular bile plug in the centrizonal area (black arrow) and occasional Mallory Denk Bodies (blue arrow) (H&E, x400); **B -** occasional focus of perivenular/pericellular fibrosis (Masson Trichrome, x200); **C -** transmission electron microscopy showing intrahepatocytic microvesicular steatosis (uranyl acetate and lead citrate - scale bar attached); **D -** transmission electron microscopy showing loss of subcellular structures (uranyl acetate and lead citrate - scale bar attached).

Various other hepatocytic changes included the occasional presence of megamitochondria, the very occasional presence of Mallory hyaline, and intracanalicular bile plugs in a centrizonal distribution (Figure 3A). Occasional perivenular/ pericellular fibrosis was noted (Figure 3B). No hepatocytic ballooning or neutrophilic satellitosis was noted. The portal tracts were normal, and no inflammation, fibrosis, or cholangiopathy was noted.

Transmission electron microscopy (TEM) performed from the formalin-fixed paraffin-embedded (FFPE) liver tissue showed the presence of intrahepatocytic fat, replacing most of the other subcellular organelles. Thus, the cytoplasm of most of the hepatocytes was devoid of any mitochondria. However, occasional mitochondria seen in the cytoplasm appeared degenerated with swollen cristae (Figures 3Cand 3D). The features seen in the liver were morphologically consistent with Alcoholic Foamy Degeneration (AFD).

The spleen weighed 145 grams. There was no splenomegaly, and the spleen showed no gross or microscopic pathology. The lungs were heavy and showed pulmonary edema. Atherosclerosis of the aorta was noted. There were no esophageal varices, and none of the other organs showed any significant pathological changes. Therefore, the final cause of death remained acute necrotizing pancreatitis, pulmonary edema, and AFD-like liver changes.

## DISCUSSION

Alcoholic foamy degeneration (AFD) is an unusual form of alcoholic liver disease (ALD) where microvesicular steatosis dominates the liver histology in the absence of the typical features of alcoholic hepatitis (AH), which it mimics clinically. First described by Uchida et al.^1^ in 1983, this uncommon entity gained recognition over time though its occurrence remains uncommon and enigmatic.^1^ We describe the characteristic histomorphology of AFD in an autopsy case of ALD.

Clinically, symptomatic individuals with AFD usually present with hepatic insufficiency. However, nonspecific clinical features can also be seen in a subset of patients.^2^ Although the initial study by Uchida et al.^1^ reported a prevalence rate of 14%, most others have reported a lower prevalence rate of 0.8%-4%.^2-3^ The variation in the prevalence rate can be partly explained by the asymptomatic cases for which the biopsies are usually not performed. These patients usually have a history of chronic alcohol consumption with binge drinking in months to weeks prior to the onset of symptoms, similar to the index case.^2,4^ AFD, due to its acute presentation, may clinically mimic alcoholic fatty liver with jaundice (AFLJ) and alcoholic hepatitis (AH), a commoner form of ALD. Clinically, the presence of neutrophilia is more commonly associated with AH and can help distinguish AFD from AH. The index case had neutrophilia, mostly associated with acute pancreatitis. AFD can show a marked increase in transaminases, especially AST, along with ALP, GGT, and bilirubin. However, AH and AFLJ can also show moderate elevation of bilirubin and liver enzymes. Besides, elevated cholesterol levels can be seen in AFD.^4,5^

Histopathologic examination is the gold standard for the diagnosis of AFD. The index case showed the classical morphology of AFD in terms of centrizonal and midzonal microvesicular steatosis (foamy change with centrally located nuclei) and cholestasis along with an admixture of macrovesicular steatosis mostly prevalent in the periportal hepatocytes. The lack of an inflammatory component, especially Mallory hyaline and neutrophilic satellitosis, is also essential for diagnosing AFD. Occasional perivenular/ pericellular fibrosis can be seen in AFD, although significant fibrosis raises a possibility of AH or AH-AFD.

Ultrastructural features of AFD are similar to the index case in the form of the presence of intracytoplasmic microvesicular fat along with disruption and disorganization of subcellular organelles, especially mitochondria and endoplasmic reticulum. Megamitochondria can be noted both ultrastructurally as well as by light microscopy. The loss/deletions of mitochondrial DNA due to alcohol leading to impaired fatty acid oxidation is the putative pathogenetic pathway of AFD that ultrastructurally corroborates with the damage to the mitochondria and endoplasmic reticulum and light microscopically corroborates with the intrahepatocytic microvesicular fat.^1^ Similar mechanism is seen in Reye syndrome, tetracycline‐induced liver injury, and acute fatty liver during pregnancy.^1,6^

Unlike patients with alcoholic hepatitis, these patients have a favorable outcome and quickly recover after alcohol abstinence. In our case, acute pancreatitis was the cause of demise, and AFD was an incidental finding on autopsy.

## CONCLUSION

We report a combination of AFD with acute pancreatitis diagnosed in autopsy in a chronic alcoholic individual. The characteristic changes in the histomorphology and electron microscopy were pivotal in clinching the diagnosis, while acute pancreatitis obscured its typical presentation.
